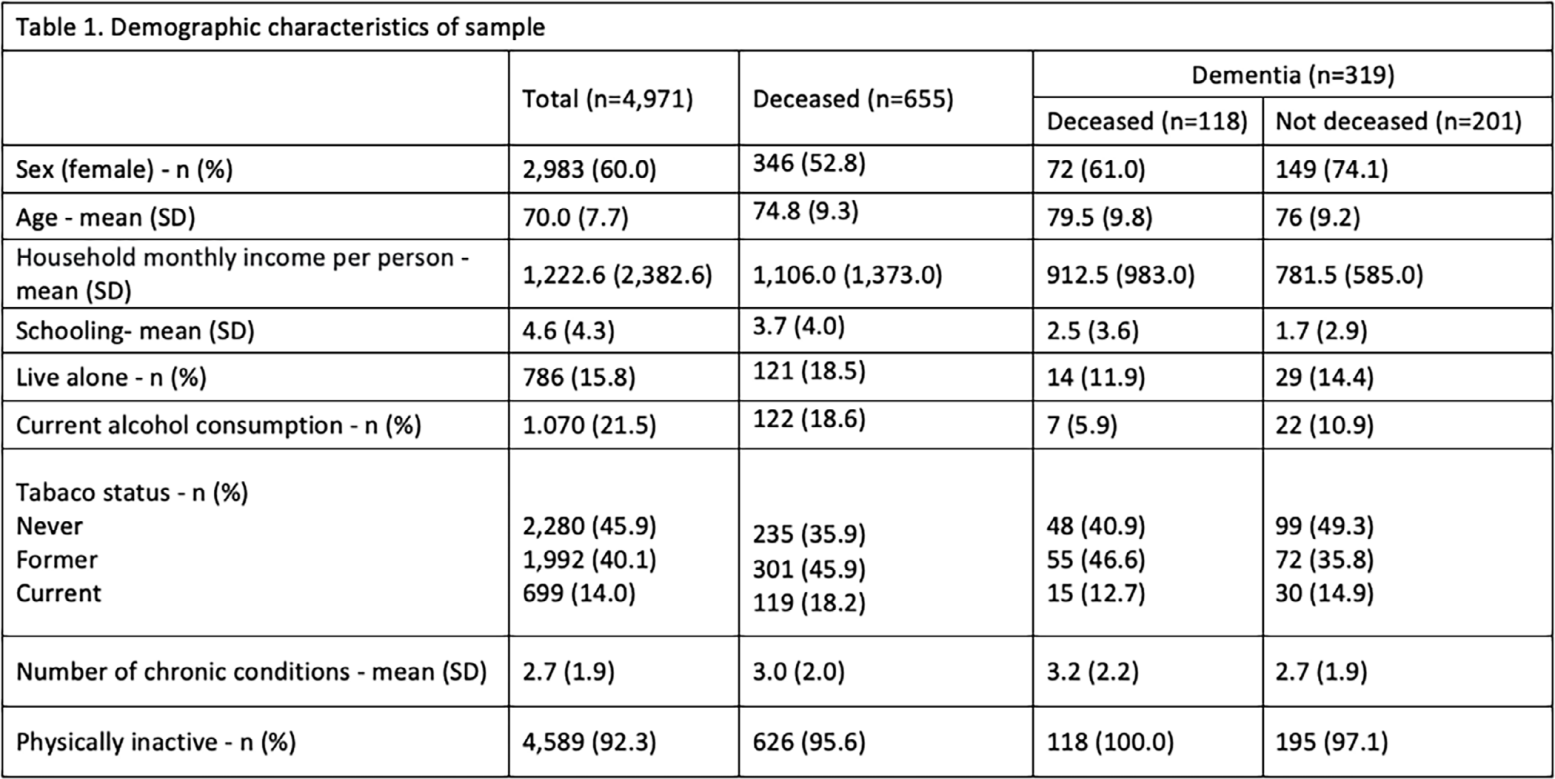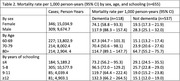# Mortality Rates in Individuals with Dementia: An Analysis of the Brazilian Longitudinal Study of Aging (ELSI‐Brazil)

**DOI:** 10.1002/alz.093343

**Published:** 2025-01-09

**Authors:** Wendell Lima Rabelo, Matheus Ghossain Barbosa, Laiss Bertola, Maria Fernanda Lima‐Costa, Cleusa P Ferri

**Affiliations:** ^1^ Universidade Federal de São Paulo, São Paulo, SP Brazil; ^2^ Universidade Federal de São Paulo (UNIFESP), São Paulo, São Paulo/SP Brazil; ^3^ Universidade Federal de São Paulo (UNIFESP), São Paulo, São Paulo Brazil; ^4^ Instituto Rene´ Rachou da Fundação Oswaldo Cruz, Belo Horizonte Brazil

## Abstract

**Background:**

Population aging is occuring faster in low‐ and middle‐income countries (LMICs) and contributes to the increasing prevalence of chronic conditions, including dementia. In Brazil, dementia‐mortality studies rely mainly on death certificate registrations, which are poorly completed, thereby compromising the results. This study examines mortality rates of older people, especially those living with dementia, in the Brazilian Longitudinal Study of Ageing (ELSI‐Brazil).

**Method:**

This is a secondary analysis of the ELSI‐Brazil longitudinal data. We estimated the prevalence of dementia using data from 5249 participants aged 60 years and older for whom the vital status was determined. We employed Cox regression to estimate the association between dementia and mortality. Mortality rates per 1000 person‐years were calculated and stratified by, sex, age, and schooling groups.

**Result:**

In the current analysis, 4971 individuals were considered after excluding those with missing data for any variable (table 1). They were primarily female (60%), with a mean age of 70.0 years (SD = 7.7), did not live alone (84.2%), and had 4.6 years of schooling (SD = 4.3). The sample comprised 4,652 individuals without dementia and 319 individuals with dementia.

In those with dementia, the mortality rate per 1000 person‐years was significantly higher in males 117.9 (95% CI: 188,3 – 157,4) compared to females 74,1 (95% CI: 158,8 – 93,3). In addition, individuals affected by dementia experienced higher mortality risks across various age groups and educational backgrounds.

The hazard ratio adjusted for multiple factors was 2.2 (95% CI: 1,8 – 2,7). Table two shows the mortality rates among people with dementia per gender, years of schooling and age‐groups.

**Conclusion:**

This study shows how dementia affects mortality in Brazil’s aging population. We expect these findings to support and direct public health interventions to improve care and support for people with dementia and their families.